# Commentary: SPG7 is an essential and conserved component of the mitochondrial permeability transition pore

**DOI:** 10.3389/fphys.2015.00320

**Published:** 2015-11-04

**Authors:** Paolo Bernardi, Michael Forte

**Affiliations:** ^1^Department of Biomedical Sciences, University of PadovaPadova, Italy; ^2^Vollum Institute, Oregon Health and Science UniversityPortland, OR, USA

**Keywords:** mitochondria, permeability transition pore, SPG7, paraplegin, cell death

SPG7 (paraplegin) is the product of the *SPG7* gene, whose mutations are responsible for an autosomal recessive form of hereditary spastic paraplegia (HSP) (De Michele et al., 1998). SPG7 is a AAA-protease (Casari et al., 1998) that co-assembles with a homologous protein, AFG3L, in the inner mitochondrial membrane. These proteins associate with unidentified proteins in high molecular weight complexes of up to 900 kDa, which are aberrant in HSP patient cells (Atorino et al., 2003; Koppen et al., 2007). Loss of this complex following deletion of *SPG7* causes decreased activity of respiratory complex I and increased sensitivity to reactive oxygen species (ROS); both events can be rescued by expression of SPG7 (Atorino et al., 2003). A recent paper suggests that SPG7 also serves an essential role in the formation and regulation of the mitochondrial permeability transition pore (PTP) (Shanmughapriya et al., 2015).

The PTP is an inner membrane channel that forms after a permissive load of matrix Ca^2+^ under conditions of oxidative stress (Bernardi, 2013; Bernardi et al., 2015). Strong evidence indicates that it derives from the F-ATP synthase, which forms channels with electrophysiological features matching those expected of the PTP in mammals (Giorgio et al., 2013; Alavian et al., 2014), yeast (Carraro et al., 2014), and Drosophila (von Stockum et al., 2015). To identify regulators of the PTP, Shanmughapriya et al. used a phenotypic screen based on the mitochondrial Ca^2+^ retention capacity (CRC) of digitonin-permeabilized cultured human cells after treatment with siRNAs designed to suppress translation of a set of mitochondrial proteins (Shanmughapriya et al., 2015). This assay (Murphy et al., 1996; Fontaine et al., 1998) is based on the assumption that the CRC reflects the state of the PTP *in situ*, i.e. its propensity to open after treatment with Ca^2+^ and PTP agonists. The screen identified 13 proteins whose suppression caused desensitization of the PTP to Ca^2+^ with an increase of the CRC. The hits included well-known modulators that do not take part in PTP formation like cyclophilin (CyP) D, the matrix receptor that mediates the PTP inhibitory effect of cyclosporin A. The Authors identified SPG7 amongst the hits, and selected it for further study because it could be co-immunoprecipitated with CyPD in a complex that also included outer membrane VDAC1 (Shanmughapriya et al., 2015). Elimination of SPG7 expression by Cas9/CRISPR methods conferred protection from Ca^2+^- and oxidant-induced PTP opening and from cell death, as expected based on PTP desensitization. The Authors conclude that SPG7 is an essential component of the PTP complex together with VDAC1, but from analysis of the results we must conclude that this is an overinterpretation that is not supported by the experimental results presented.

First and foremost, the phenotypic screen does not allow a distinction between core PTP components from regulators. This difference–only core component of the PTP must necessarily be essential to PTP formation while regulators may only modulate PTP activity- represents important and mechanistically discrete phenomena. Indeed, failure to appreciate this critical difference has often confused our understanding of the molecular composition of the PTP. In this study, the results show that the PTP opened, albeit at higher Ca^2+^ loads, after suppression of all 13 transcripts including SPG7 (Shanmughapriya et al., 2015). Thus, the PTP opens even in the absence of SPG7, much as it does in the absence of CyPD (Baines et al., 2005; Basso et al., 2005; Nakagawa et al., 2005; Schinzel et al., 2005) questioning the conclusion that the protein is an essential component of the pore. Second, the mammalian PTP displays conductances up to 1.2 nS (Szabo and Zoratti, 2014) that are unlikely to be generated by the 2-transmembrane domain proteins SPG7 and AFG3L. Indeed, and in spite of its claims, the study of Shanmughapriya et al. does not address the question of whether the putative “PTP complex” formed by SPG7, AFG3L, and VDAC1 can actually form channels at all. Thus, the graphical abstract depicting the PTP as a complex of SPG7, AFG3L, and VDAC1 is a misrepresentation of the actual findings of the paper and of the literature on the PTP. The putative role of VDAC1 deserves a specific comment.

VDAC1 is the major outer membrane protein and one of the most abundant mitochondrial proteins in mammals. Its association with the PTP was suggested based on co-purification with other putative components, i.e. the adenine nucleotide translocase and the peripheral benzodiazepine receptor, today called TSPO (McEnery et al., 1992). The link was made because ligands of TSPO are also agonists of the PTP (Kinnally et al., 1993). As shown by experiments on mitochondria from mice where the corresponding genes were deleted, neither TSPO (Šileikyte et al., 2014) nor VDAC1 (Krauskopf et al., 2006) is an essential component of the PTP or a regulator of its activity, and the effects of “TSPO ligands” on the pore could rather be explained by their interaction with the F-ATP synthase (Cleary et al., 2007; Giorgio et al., 2013). Of note, also genetic inactivation of the less abundant VDAC2 and VDAC3 isoforms does not affect PTP opening and PTP-dependent cell death (Baines et al., 2007). Thus, the reported co-immunoprecipitation of SPG7 with VDAC1 (Shanmughapriya et al., 2015) does not bear on the nature or regulation of the PTP. Our comment does not imply that the outer mitochondrial membrane does not regulate PTP activity, as discussed in detail (Bernardi et al., 2015).

Surprisingly, Shanmughapriya et al. do not discuss possible mechanisms through which SPG7 may regulate the PTP. We suspect that the high molecular weight complex formed by SPG7 and AFG3L reported by Casari and coworkers (Atorino et al., 2003; Koppen et al., 2007) may be due to a direct interaction of the AAA-protease heterodimers with F-ATP synthase, which may in turn stabilize dimers/oligomers of the complex and thus favor Ca^2+^-dependent PTP formation (Bernardi et al., 2015). Increased oxidative stress due to inhibition of complex I could easily explain sensitization of the PTP. Thus, SPG7 could be one more of the many regulators of the PTP, but not an essential component of the pore.

## Conflict of interest statement

The authors declare that the research was conducted in the absence of any commercial or financial relationships that could be construed as a potential conflict of interest.
